# A Review of the Effects of Major Atmospheric Pollutants on Pollen Grains, Pollen Content, and Allergenicity

**DOI:** 10.1155/2015/940243

**Published:** 2015-12-24

**Authors:** Hélène Sénéchal, Nicolas Visez, Denis Charpin, Youcef Shahali, Gabriel Peltre, Jean-Philippe Biolley, Franck Lhuissier, Rémy Couderc, Ohri Yamada, Audrey Malrat-Domenge, Nhân Pham-Thi, Pascal Poncet, Jean-Pierre Sutra

**Affiliations:** ^1^Allergy & Environment Team, Biochemistry Department, Armand Trousseau Children Hospital (AP-HP), 26 avenue du Dr. Arnold Netter, 75571 Paris, France; ^2^Physical Chemistry of Combustion and Atmosphere Processes (PC2A), UMR CNRS 8522, University of Lille, 59655 Villeneuve d'Ascq, France; ^3^Pneumo-Allergology Department, North Hospital, 265 chemin des Bourrely, 13915 Marseille 20, France; ^4^Persiflore, 18 avenue du Parc, 91220 Le Plessis-Pâté, France; ^5^CNRS, 75794 Paris 16, France; ^6^SEVE Team, Ecology and Biology of Interactions (EBI), UMR-CNRS-UP 7267, University of Poitiers, 3 rue Jacques Fort, 86073 Poitiers, France; ^7^KeyGene, P.O. Box 216, 6708 AE Wageningen, Netherlands; ^8^Biochemistry Department, Armand Trousseau Children Hospital (AP-HP), 26 avenue du Dr. Arnold Netter, 75571 Paris 12, France; ^9^French Agency for Food, Environmental and Occupational Health Safety, 14 rue Pierre et Marie Curie, 94701 Maisons-Alfort, France; ^10^Allergology Department, Pasteur Institute, 25-28 rue du Dr. Roux, 75724 Paris 15, France; ^11^Infections & Epidemiology Department, Pasteur Institute, 25-28 rue du Dr. Roux, 75724 Paris 15, France

## Abstract

This review summarizes the available data related to the effects of air pollution on pollen grains from different plant species. Several studies carried out either on* in situ* harvested pollen or on pollen exposed in different places more or less polluted are presented and discussed. The different experimental procedures used to monitor the impact of pollution on pollen grains and on various produced external or internal subparticles are listed. Physicochemical and biological effects of artificial pollution (gaseous and particulate) on pollen from different plants, in different laboratory conditions, are considered. The effects of polluted pollen grains, subparticles, and derived aeroallergens in animal models, in* in vitro* cell culture, on healthy human and allergic patients are described. Combined effects of atmospheric pollutants and pollen grains-derived biological material on allergic population are specifically discussed. Within the notion of “*polluen*,” some methodological biases are underlined and research tracks in this field are proposed.

## 1. Introduction

Atmospheric pollution has to be considered nowadays as one of the main characteristics of areas where, worldwide, human population density is at high level. From the middle of the 19th century to now, its increase is huge in some continents and very important in many others. Heede recently asserted that the highest anthropogenic carbon dioxide and methane emissions originated from less than 100 commercial and state-owned entities, worldwide, from 1854 to 2010 [1]. The atmospheric pollution by gases and particulate matter affects many rural, industrial, and urban sites [2–4]. In the last report of the European Environment Agency, it is possible to read that up to 96% of the European Union's urban population is currently exposed to fine particulate matter concentrations above World Health Organization guidelines [5]. It is thus undeniable that atmospheric pollution plays a major role in all age groups' health [6–10].

In addition to pollutants gases and particles emitted consecutively to human activities, the atmosphere is the medium of transit for a wide variety of biogenic particles. Among biogenic particles, the bioaerosol consists of very different types of particles like viruses, bacteria, mold, plant fibers, or pollen with a broadly open size distribution from tens of nanometers to a few hundred micrometers. It is known for quite long that these particles are very active for very diverse potential diseases [11–15]. Among them, pollen grains are a real concern in allergy [16]. If pollen is one of the main agents in the reproduction system for thousands of plant species, from Gymnosperms to Angiosperms, it is at the same time a real contributor of the total bioaerosol mass. Based on the concentration of phospholipids, Womiloju et al. reported that cell materials of fungi and pollen could contribute 4–11% of the total particulate matter < 2.5 *μ*m (PM_2.5_) mass and 12–22% of organic carbon in fine particulate matter (PM) [17]. It is also the direct carrier of water- and non-water-soluble allergens as well as the vector for outer and inner subparticles for which various allergens were recently characterized. When broken, the pollen grains of anemophilous plants are also the vectors of fragments. Many of these fragments are able to diffuse airborne allergens as can do innate pollen subparticles [16] https://www.anses.fr/sites/default/files/documents/AIR2011sa0151EN.pdf. It has already been written that the most frequent aeroallergens derive from pollen [18]. Some of the effects of pollution on this specific fraction of the global bioaerosols are already known and reviewed [19–31]. What is proposed here is focusing on the diversity of approaches that were chosen in order to study, at different scales and for different pollutant sources, year after year and decade after decade, the various links between atmospheric pollution, airborne pollen, allergenicity, and allergy. Through updated literature, this review aims also to take into account both the potential methodological biases and different tracks for new more standardized research way in the aerosol-allergy field.

## 2. Effects of Pollutants on Pollen Grains and Pollen Content

Atmospheric pollutants may have the following direct effects on pollen: (a) modifications of their biological and reproduction functions: decrease in viability and germination, (b) alteration of the physicochemical characteristics of the pollen surface, (c) change in the allergenic potential, and (d) adjuvant effect increasing their potential health hazards. To experimentally investigate these effects and depending on scientific goals, pollen may be either fumigated in laboratory facilities with artificially generated pollutants or directly exposed to outdoor atmospheric pollution.

### 2.1. Pollen Exposure in Outdoor/Field Experiments

#### 2.1.1. Localization and Experimental Conditions

Two main groups of field experiments on polluted pollen material can be defined. In the first one, the research is done on collected pollen (passive exposure) and collected particles from pollen grains. In the second one, some pollen material is exposed in different polluted zones within a specified period (transplant method). Passive exposure has for main advantage to provide a realistic pollution pattern and to integrate the short and long term effects of pollutants on plants. A lot of pollutants may interact with pollen and plants, including soil pollution, and in this kind of studies the real exposure is very difficult, even impossible, to evaluate. Confounding factors are other important parameters to keep in mind, including and not limited to sunlight exposure, health and individual (genetic) susceptibility of plant, and wet and dry deposition. Transplantation method has the main advantage of eliminating confounding factors due to heterogeneities in the history of the plant, exposure, or individual susceptibility [35–37].

*(1) Collected Material*

*(i) Whole Pollen Grains*. Whatever the geographical locations, different approaches were proposed. The most common is the comparison scheme in which the material to be studied is collected both in a polluted zone (town center and road/highway sides) and in a control zone (rural suburbs in many cases and mountainous zones) [38–40]. Some other studies focused on zones known for their different levels of regularly measured pollution in order to compare the pollen material at the different polluted sites [41, 42]. Transects were also used, for instance, along a specific road [43] or according to the altitude [44, 45]. In different continents, some authors investigated forest zones [46–51], sometimes under specific sophisticated ecological protocols [52] including the simulation of acid rain on selected trees from experimental orchards before the collect phase of pollen material [53]. Among other forest studies, a comparison of polluted versus nonpolluted populations was proposed. Using polluted pine pollen (*Pinus sylvestris*), the potential influence on pollen vigor and then seed yield produced in nonpolluted zones where polluted pollen can be transported for long distance has been investigated [54]. Finally, some research propositions chose a set of sampling places from 10 (around a factory producing copper, nickel, sulfuric acid, and fertilizers) [55] to larger than 10, for instance, the study by Armentia et al. [56] or the one by Citterio's team from Milano on the Lombardy Po river plain region (Italy) [57]. The choice of plants is very large, from ornamental plants [58–60] to different trees [39], the pollen of which being known as allergenic or not. Nevertheless, a relatively high percentage of studies focused on a reduced number of pollen grains like allergenic* Betula*,* Ambrosia*, and grass ones in Europe, whereas in Japan the interest was often on* Cryptomeria* and/or other Cupressaceae which are locally among the major allergenic pollen sources. Such studies on collected pollen are the most numerous regarding field experiments.

*(ii) Pollen-Derived Particles and Free Allergens*. Several research groups could experiment, in urban polluted air, on detection of subparticles such as aeroallergens issued from weed [57] and tree [61] pollen. When analyzing the ambient air samples, it was possible to show that some airborne antigens were mainly adsorbed to combustion particles (soots) that have a very large surface-to-volume ratio. They can thus fly as depots on diesel exhaust particles (DEP) generated from transportation [62]. They can also be associated with the presence of alkaline rainwater in polluted zones (experiments on atmospheric Cry j 1 from* Cryptomeria japonica* pollen) [63]. The technical device used for such detection combined, for instance, (i) Andersen high-volume sampler allowing collecting, on quartz fiber filters, size-segregated particles from less than 1.1 *μ*m to 7 *μ*m and (ii) surface plasmon resonance (SPR) technology enabling through monoclonal antibodies binding to ensure atmospheric allergen detection.

*(2) Exposed Pollen*. In this specific kind of experimental design, the source pollen material is exposed both in polluted zones and in control zones in small bags (made, for instance, with either polyester thread or nylon) or other container-like equipment. The purpose is to get a semiquantitative value of the direct effect of measured global pollution on a known pollen powder weight. Such experiments were proposed in Europe on* Betula*,* Fagus*, and* Dactylis* pollen [41, 64–68] and sometimes at different places [69] (for instance, comparison of 2 different towns). In some Middle East regions, studies on exposed pollen were performed on herbs and trees [58, 59, 70, 71].

#### 2.1.2. Physicochemical Effects of Atmospheric Pollution on Pollen

*(1) External Surface of Grains*. Different effects of pollution on external surface of exine were demonstrated by several teams on collected as well as on exposed pollen material. They used mainly light microscopy as well as scanning electronic microscopy (SEM) and transmission electronic microscopy (TEM). The most prominent results in many experiments show that exposure to the ambient air pollution increased the fragility of exine. It causes collapse and numerous cracks in its surface according to the initial fragility of this specific external pollen membrane. For instance, Cupressaceae pollen exines, rather thin [72], are clearly more easily fragilized [39, 73, 74] than some other pollen grains from other plant species/families. Regarding airborne PM, Chehregani and Madj's team showed that a large part of it can accumulate on the surface of pollen grains and change the shape and tectum of pollen [58, 75] what was also shown on exposed pollen (*Dactylis glomerata* and* Betula verrucosa*) in the Mulhouse town experiments [67, 68].

When comparing pollen (*Chenopodium album*) from northern Portugal's rural zone to some other materials from the city of Porto, Abreu's team could demonstrate that the first one has opercula well defined, while in the urban pollen there is a fine film covering its wall and opercula are deformed. Through micro-Raman spectroscopy analysis, it was possible to evidence that some carbon-containing particles are adsorbed and accumulated on the surface of the studied material [76]. Amjad and Shafighi showed also surface deposits on* Chenopodium album* collected pollen under polluted conditions [77]. But even on light microscopy (400x), Adhikari et al. reported finding* Ambrosia* pollen grains covered with black particulate matter in the analyzed airborne material from Cincinnati (OH, USA) [78].

*(2) Elemental Composition*. When studying the elemental composition, the analytic device used was, for instance, made of different combined techniques like energy dispersive X-ray (EDAX), secondary ion mass spectrometry (SIMS) imaging, electron spectrometry for chemical analysis (ESCA), and also emission electron probe microanalyzer (EPMA) [79–81]. If, in terms of mineral composition, K is the dominant element in freshly harvested pollen from anemophilous plants [69, 82], several other mineral ions can, of course, also be found [73]. Among various metal trace elements (MTE), at various sampling zones in Stockholm, increased quantities of Zn were found for the polluted* Betula* pollen (SIMS experiment) [66]. Differential concentration of Pb in pollen is attested in material from Compositae according to its presence in zones where the pollen was collected [83]. Apart from Zn and Pb, Cu was also investigated on several Gymnosperm and Angiosperm trees by Cox in relationship with pH changes [47]. Nevertheless, from a screening of different studies [71, 73, 79, 81, 84], it seems difficult to find convergent changes in the modification of elemental composition (polluted versus unpolluted), even if, as mentioned by Oleksyn et al. on* Pinus sylvestris* forest population, enhanced accumulation by pollen of such elements as Al, Mn, Cu, Ni, and Cd, for instance, may adversely affect pollen function [51].

*(3) Chemical Compounds Adsorbed on Grains*. An increase in concentrations of total flavonoids (HPLC analysis) is detectable when comparing pollen (*Thuja orientalis*) from polluted and unpolluted sites [39]. Rezanejad suggested that some plant defense mechanisms initiated higher flavonoids biosynthesis in pollen affected by airborne particulate matter. In the Kanto region (Greater Tokyo Area, Japan), Wang's team showed on another pollen from the same Cupressaceae family (*Cryptomeria japonica*) that the amount of ionic components of both particles (NO_3_^−^, SO_4_^2−^, and NH_4_^+^) and gaseous pollutants (NO_2_, SO_2_, and NH_3_) deposited on perigonium and pollen grains was higher in urban polluted zone than in mountainous areas [40].

#### 2.1.3. Effects of Outdoor Atmospheric Pollution

*(1) Pollen Biological and Reproduction Functions*. The various studies carried out, mainly from the end of the 1960s to the 1980s, on the effects of acid rains on different tree pollen from European and American forests, were before all designed in order to know more about the pollen germination and viability under polluted conditions. The forest's health and its reproduction were thus at the very center of the investigations. Even if forest-oriented and/or agro-oriented, such research ways, still recently active [52], give elements that can help achieve a better understanding on pollen content changes and may be also on the most significant proteins involved in such viability changes, some of which could be implicated in allergy diseases.

In the “exposed pollen” part of the Mulhouse town (France) experiments [80], the viability and germination tests clearly showed that the freshly harvested pollen material lost totally its biological and reproduction functions in industrial and high-traffic road zones after 2 full days of exposure for* Betula verrucosa* and* Dactylis glomerata* pollen (control, resp., 70% and 87.5% viability). It is not the case for* Fagus sylvatica* pollen material (68% in high-traffic road zones, 45% in industrial one, and 79% for the control). A decrease in viability and/or germination has been consistently observed for outdoor-polluted pollen of various species:* Pinus pinea* [85],* Pinus nigra* Arnold [42], and* Pinus sylvestris* [48, 54, 86],* Betula verrucosa* [80] and* Betula papyrifera *[87],* Hedera helix* L.,* Convolvulus sepium* L.,* Cynodon dactylon* (L.) Pers.,* Quercus ilex* L.,* Dactylis glomerata* L.,* Parietaria diffusa* M. and K.,* Daucus carota* L.,* Tilia cordata* Miller [88],* Corylus avellana* L., and* Rosa rugosa* [89].

Even if viability, germination, and allergenicity of pollen grains are not necessarily intercorrelated [90] or simply not fully understood for the moment, it is, for instance, known that, at least for grasses pollen, some proteins like the group-1 grass pollen allergens (*β*-expansin) play a major role in the reproduction process of these very numerous herbs. Zea m 1 has indeed a large effect on pollen tube growth rates* in vivo* [91]. It is also known that formation of reactive oxygen species (ROS) in pollen starts at the early germination stage, before the formation of the pollen tube, generated mainly by NAD(P)H oxidases, in insoluble fractions [92–95]. Thus pollen viability, pollen germination, allergenicity, and air pollution effects on pollen could have some important common points.

*(2) Protein Modifications*. Different modifications induced by air pollution were evaluated at the protein level. Pollutants can play a role in the variation of protein amount and/or the presence or absence of proteins bands from comparative extracts. For instance, Shahali et al. found a decrease in total protein amount on polluted sites' pollen (*Cupressus arizonica* in Tehran region) as well as a net decrease of Cup a 1 [73]. In a study on rural versus urban pollen, Guedes et al. observed differences in protein profiles since bands of 16 and 36 kDa (from* Chenopodium album* water-soluble pollen extract) disappeared in the pollen collected from more polluted area [76]. Madj's team also found such protein feature (some protein bands disappearing pattern) when comparing pollen from polluted versus nonpolluted zones for pollen material from different plant species [75].

*(3) Allergens Balance and Composition Changes*. Regarding the specific proteins called allergens and the modifications induced by air pollution on collected or exposed pollen as well as on pollen-derived material, results are not fully convergent. Some studies like the one developed in Finland along transect did not detect any differences in birch pollen allergenicity according to the studied pollution gradient. The authors indicated then that combined sulfur dioxide and heavy metal pollution do not affect pollen allergens [55]. Some other studies, also on* Betula* pollen, when exploring urban versus rural areas' pollen extracts using comparative electrophoresis experiments (DIGE) could reveal 26 differences in protein spot intensity of pollen of the two sampling zones. One of these proteins was identified by* de novo* sequencing as a 14-3-3 protein, which resembles a stress-induced factor in other plant species [96]. The allergen content of exposed* Betula* pollen (Mulhouse experiments) was noticeably different from the one obtained with clean control pollen [67]. Recently, at the ZAUM (Munich), Traidl-Hoffmann's team found an enhanced allergen content of polluted birch pollen extracts when comparing pollen of both low O_3_-exposed trees (54 *μ*g/m^3^) and high O_3_-exposed trees (85 *μ*g/m^3^). An altered composition of adjuvant pollen associated lipid mediators (PALMs) [97], among which are E1-phytoprostanes, was observed with this enhancement of allergenic content [98]. Such results on ozone pollution effect on* Betula* pollen grains go in the same way as what was reported by another German team 15 years ago on grass pollen [99], showing the importance of O_3_ as current pollution source of city pollen. In Mediterranean towns and surroundings, some allergens like Cup a 3 (thaumatin-like protein) are mainly expressed in cypress pollen suffering stress condition, for instance, air pollution, because of heavy traffics [100]. By contrast, in pollen obtained from a garden with a low pollution, this protein was not expressed [101]. A rather exhaustive study on* Cupressus sempervirens* pollen proteome and allergome did not detect Cup s 3 (the equivalent of Cup a 3 for this species) on unpolluted pollen [102]. In another work on pollen from a Gymnosperm species (*Pinus radiata*), a Spanish group found again O_3_ effects [103]. From three different sites, (i) from rural sites, (ii) from road site (with healthy trees), and (iii) again from road side but with trees infected by a fungus (potentially more plant defense proteins), pine pollen was collected and several atmospheric pollutants measured (NO_2_, NO, NO_*x*_, O_3_, PM_10_, and SO_2_). The pollen extracts from these 3 sites material were tested on a pool of 10 sera from 35 selected pine pollen-allergic patients submitted initially to skin prick tests (SPTs). The highest levels of specific IgE were found with the extract from the rural zone in which O_3_ levels (45.9 *μ*g/m^3^) were the highest, with lower values for other pollutants. No significant differences were detected in immunoblotting experiments [103]. It could thus be concluded that pine pollen allergenicity increased when trees are under elevated O_3_ conditions.

When comparing* Lolium perenne* pollen extracts from polluted urban zones versus unpolluted rural zones in and around Valladolid (Spain), Armentia et al. (2002) showed a significant difference in the skin reactivity of the 20 urban and 20 rural tested patients to the extracts, with a greater response regarding the urban pollen source. The highest concentration of Lol p 5 (3,35 *μ*g per gram of pollen) was detected in “urban” pollen extracts [56].

#### 2.1.4. Dispersion of Subpollen Particles

The presence of subpollen particles, innate ones and issued from fragmented pollen grains, is attested for long time [104]. From species to species, these particles are nonhomogeneous in size [105, 106] and nature [107–111]. Their implication in allergy diseases is already described in several studies for different regions and pollen sources [112–121]. Their presence in the atmosphere increases the bioavailability of pollen allergens. The role of pollution on release and dissemination of these subparticles is documented. Regarding the effects of pollutants on the allergenicity of this specific airborne material itself, the research stays reduced, even if, for instance, different Japanese groups provided in the last 10 years very accurate work.

*(1) Outer Subparticles*. The innate outer subparticles are known for pollen from different plant species among which Taxodiaceae and Cupressaceae ones are the most common in atmospheric material [30, 122]. These orbicules, also called Ubisch bodies, [123–127] are of small size (0.5–2 *μ*m) and thus have lower sedimentation speed compared to pollen grains. Some effects of air pollutant studied on* Cryptomeria japonica* pollen, as shown by Wang's group, are morphological changes and production of, taking a term from physics, “daughter” allergenic particles [128–130]. Wang et al. used the term of “transmutation” in order to describe the modification from source (trees from mountainous areas) to deposition zones (urban sites) [40]. Allergenic Cry j 2 from* Cryptomeria* is attested from pollen wall surface of outer subparticles when using appropriated sampling and SPR analysis for the detection [131].

*(2) Inner Subparticles*. The same Japanese research group developed successfully the detection of Cry j 2, in starch granule material in the polluted* Cryptomeria* pollen grains, with the same type of device (Andersen high-volume air sampler associated with SPR allergen detection). In the multisites Lombardy region (Italy) experiment, the percentage of inner subparticles from* Ambrosia* pollen source was not significantly higher in pollen samples collected along high-traffic roads than in those collected in vegetated areas [57]. Moreover, Shahali et al. [74] observed an increased fragility of the exine that may facilitate the inner particles liberation.

### 2.2. Laboratory-Generated Pollution

If studying the effects on pollen of major pollutants in “real” atmosphere is of high importance, it stays often difficult to provide clear ideas regarding the pollen and pollution interactions because of the multiple components mixed in the different kinds of nowadays pollution existing in various parts of the world and because finding a single experimental device allowing approaching the multiple interactions pollen-pollutants is just, currently, not possible.

Thereby, choosing the way of controlled experimental pollution in either laboratory or greenhouse conditions sounds quite sensible. It can provide, for different gases, selected mineral versus biogenic particulate matter, alone or combined, a clearer appreciation for each pollutant. Such way gives the possibility to measure time, dose, and quantity. It allows the repetition, for various pollen from different plant species and/or pollen subparticles, of multiple pollution scenarios at low, realistic, or high pollution rates, emphasizing, for instance, pollution mimicking industry or traffic conditions, western life, or developing countries contexts.

#### 2.2.1. Physicochemical Effects of Artificial Pollution

*(1) Modification of the External Surface*. The contact gas-pollen [132] and/or PM-pollen may have an effect on the most external part of the exine [69, 133–135]. The design and use of fluidized bed reactors dedicated to the pollen-pollution interactions by Behrendt's group in the 1990s gave the opportunity to study these interactions at different levels with a great accuracy. If they could find, as did other authors, that pollen surface is covered with atmospheric particles when using a specific dose of airborne PM [136], moreover, Behrendt et al. showed that there is morphological evidence for preactivation of pollen by organic extracts of airborne particulate matter [137]. Under some conditions, aqueous compounds may then induce local allergen release, resulting in either allergenic extrusions followed by generation of allergenic aerosols or adsorption of pollen-derived proteins to airborne particles [138].

Some authors found a deep physical modification of pollen exine with artificial pollution, whereas others found no differences between polluted and nonpolluted pollen. For example, pollen of* Platanus orientalis* became swollen after several hours of fumigation with NO_2_, SO_2_, or NH_3_ [139]. In another study where* Glycine max* L. plants were exposed to atmospheric relevant concentration of CO_2_ [140] collapsed pollen grains were observed without apertures and with a disturbed exine ornamentation. In another study using plants fumigation (*Lolium perenne* L.), the number of underdeveloped pollen grains was higher in ozone-exposed plants. On the other hand, Ruffin et al. found no significant pollen morphologic changes despite the use of very high doses of pollutants (1% of NO_2_, SO_2_, or CO) [141]. Kanter et al. found no physical modification of ragweed pollen from plants exposed to 80 ppb of ozone during the entire vegetation period [142] and Lhuissier et al. found no structural damage to* Betula* and* Dactylis* pollen material with very high doses of CO [143] conflicting with the results of Cerceau et al. where collapsed* Betula* grains were observed with CO [69]. The source of these discrepancies comes probably from differences of pollen used and also the variable doses of gas pollutants. One of the major constituents of pollen, water, is indeed rarely documented in pollen/pollution studies. By changing shape and size of pollen, water content will probably play a role in physical properties of the exine. Depending on gathering period and meteorological and stocking conditions the water content of pollen grains will be profoundly affected. In their work on acidic species adsorption onto pollen grains, Okuyama et al. concluded that the uptake of water-soluble gaseous species will be promoted by the moisture on the pollen surface [144]. More generally, humidity during the pollen fumigation appears to be a very important parameter regarding pollen viability/germination studies [27]. Despite the ease of its determination and its potential importance, the water content of the pollen grain is very rarely studied and there is an almost systematic lack of published work. Moreover, Okuyama et al. studied the acid adsorption properties of the pollen. One of the conclusions was that nitric acid is not only adsorbed on the surface but also dissolved into the inner part of the pollen, changing thus its chemical balance [144].

Interestingly, Motta et al. observed damage to* Phleum pratense* pollen grains with NO_2_ but not with ozone under the same experimental conditions [145]. This result showed that pollen of same species had different tolerance to different pollutants. The lack of systematic studies with pollen from different plant species exposed to the major pollutants in the same experimental conditions is a striking fact. It has to be mentioned however that such studies will be very difficult to pursue as the main obstacle will be the collection of freshly gathered pollen from large amount of different geographical localization and all seasons.

*(2) Elemental Composition and Chemical Changes*. Elemental composition of pollen is very often modified by pollution. Indirect proof of chemical changes was in fact observed in fluorescence spectra of pollen [146–148] and elemental composition was also determined in a few laboratory experiments but without a clear convergence on the chemical modifications observed [69, 149]. In a recent series of experiments that tried to compare the effects of pollutants on pollen from different plant species as what was proposed previously by Ruffin et al. on 3 trees (*Quercus*,* Pinus*, and* Ulmus*) and a grass (*Festuca*), NO_2_-exposed pollen [141], Visez and his team, using a specific device, could show that the uptake of NO_2_ is not the same according to the analyzed pollen. The susceptibility of pollen to NO_2_ effects is decreasing as such: cypress, timothy grass, and finally birch [150]. Pollen modifications due to O_3_ have also already been shown to be species-dependent by Ribeiro et al. [151].

Laboratory studies on the kinetics and extent of the interactions of pollution, in both gas and particulate phase, are relatively scarce [152]. The timing of pollen pollution is not known. In our current state of knowledge, the amount of pollution suffered by the pollen directly in the plant cannot be indeed associated with the modification during the comparatively short pollen airborne life.

#### 2.2.2. Biological Effects of Artificial Pollution

*(1) Pollen Viability*. Rate of germination and viability are the simplest biological parameters to demonstrate the effect of pollution on the pollen. Thus, a great number of studies report the influence of artificial pollution on the pollen reproductive function for a wide variety of plant species and a range of common atmospheric pollutants and concentrations: carbon monoxide, nitrogen and sulphur dioxide, ozone, acids, and heavy metals (see the review by Wolters and Martens [27]).

For example, when artificially polluting* Ambrosia artemisiifolia* pollen with O_3_, Pasqualini et al. could clearly show that the viability was rather low on 7-day O_3_^−^ fumigated pollen (39%) compared to the control sample (56%) [157]. On pollen of cultivated plants (tomato, tobacco, and petunia), Feder showed that the pollen viability can be reversibly slowed or stopped by exposure to low concentrations of O_3_ [158]. O_3_ effect can also be observed in* Lolium perenne* pollen. Development was disrupted by inhibiting starch accumulation throughout the anther [159]. The impact of fumigated O_3_ was also significant on* Brassica napus* (and different from the one by SO_2_) in a greenhouse study carried out by Bosac et al. [160]. As for field studies on forest plants trying to understand the effects of acid rain, the role of pollutants in specific tree pollen grains has also been studied experimentally. Simulated acid rain showed real effects on* Picea* pollen [161], but it was demonstrated that broad leaved tree pollen was more sensitive than conifers, with an intermediate sensitivity for the understory species [162].

Pollen tolerance to pollution seems to be higher when the grains are exposed* in vivo* (directly on the anther) compared to* in vitro*, pointing to a protective role of the anther. Sensitivity of pollen is enhanced when exposure occurred* in vitro* directly in the germination medium [27]. For some species, a decrease of germination consecutive to ozone pollution has indeed occurred only in condition where gaseous or liquid water was added [43, 160]. Most commonly, the pollen viability is diminished, even suppressed, consequently to an artificial pollution event and the decrease is often proportional to the pollutants dose. Rates of germination were also negatively affected for several species exposed* in vitro* with very low doses of pollutants: O_3_ (30 ppb/6 hours) and NO_2_ (34 ppb/6 hours) [153, 156, 163].

Given the diversity of pollutants, plant species, and fumigation methods employed, no quantitative trends can be given but the general following conclusions can be drawn:

- Atmospheric pollution, in a broad sense, generally induces a decrease in the pollen viability and germination.
- Anthers have a protective role.
- Very low doses of pollutants may have harmful effects.

*(2) Total Proteins and Allergens Modifications*

*(i) Laboratory Conditions*. In a series of simulations, after incubating pollen from rye, birch, and ash tree with atmospheric dust and similarly exposing the grains to SO_2_, NO_2_, and O_3_, Thomas et al. could show a release of protein material. The release was the most important with the most concentrated rate of dust [164, 165]. In the same kind of work on protein content, Bist et al. found a significant decrease in the protein concentration in* Ricinus communis* pollen exposed to NO_2_ individually and those exposed to SO_2_ and NO_2_ together [166]. A convergent pattern was documented in the studies by Santra et al. and Parui et al. on the protein content of, respectively, fumigated (SO_2_ and NO_2_) pollen grains (*Cocos nucifera* and* Datura metel*) and SO_2_-exposed pollen grains (*Argemone mexicana*) that changed with increased exposure time and concentration of relevant gases [167, 168]. Pollen from 3 Betulaceae species (*Betula pendula*,* Ostrya carpinifolia*, and* Carpinus betulus*) exposed to NO_2_ showed also total soluble proteins decrease when compared with the nonexposed pollen [153]. On CO-treated* Betula* pollen, Lhuissier et al. documented a decrease of 70 to 40% in the water-soluble proteins amount (CO concentrations ranging from 0.01 to 5%) [143]. With SO_2_-exposed pollen (*Acer negundo*) samples, Sousa et al. could document lower protein content compared with the control sample [169]. Not only on the* Acer negundo* pollen but also on* Quercus robur* and* Platanus* spp. ones, the same Portuguese group, in a first paper [170], could detect significant O_3_ effects through a decrease in soluble proteins for the 3 species with an exposure at 58 ppb, with the O_3_ limit value of the European Union Directive 2008/50/EC (2008) on ambient air quality being 61 ppb. In a second paper [151], the values of protein concentrations were coherent for both* Platanus* and* Quercus* pollen according to previous results (resp., 4,380 *μ*g/mL, control, versus 3,150 *μ*g/mL, O_3_-exposed material, and 4,575 *μ*g/mL, control, versus 4,185 *μ*g/mL, O_3_-exposed material) but not for* Acer* with a slight increase (5,783 *μ*g/mL, control, versus 5,834 *μ*g/mL, O_3_-exposed material). According to the authors, the heterogeneity seems linked, at least for some changes, to differences in pollutants (SO_2_ versus O_3_) and interspecies variations. The choice of material and initial treatments could also be evoked.

Different works on pollen allergenicity evaluated modifications of the allergen content by O_3_ fumigation. On* Ambrosia artemisiifolia* SDS-PAGE profiles produced in Pasqualini et al.'s work, 38 kDa protein band has been detected which corresponded to Amb a 1, the major antigenic component of ragweed pollen, but without any quantitative difference between nonfumigated and O_3_-fumigated pollen grains. In this study, the 7-day O_3_ fumigation did not induce changes in the content of the major allergen Amb a 1 [157]. Some other groups carried out experiments that are nonconvergent with these results. Thus, exposure of rye (*Secale cereale*) cultivars to elevated O_3_ levels increases the allergen content in pollen [171]. In the already cited paper from Porto's group [151], O_3_ effects differed depending on the plant tested. In* Acer negundo* and* Quercus robur* pollen, from one-dimensional (1D) SDS-PAGE immunoblot experiments, an increase in IgE reactivity (in the majority of the tested sera) or no differences were observed in O_3_-exposed samples compared with nonexposed pollen, while in* Platanus* pollen nonexposed pollen leads to higher IgE reactivity compared with the exposed samples.

The effects of NO_*x*_ and CO through electrophoretic analysis, immunoblots, and ELISA were important on protein contents and on the recognition of* Dactylis* and* Betula* pollen allergens by IgE from allergic patient sera. These effects were not genus-convergent [155, 178]. Rogerieux, within Peltre's team, could find, when using high levels for the 3 different experimental gases, SO_2_ (1,300 *μ*g/m^3^ for 1 h, i.e., 7 times the warning level of European guidelines), NO_2_ (950 *μ*g/m^3^ for 1 h, i.e., again, 7 times the European warning level), and O_3_ (200 *μ*g/m^3^ within the European warning level), 3 types of results. These results were (i) no significant difference for air-exposed* Phleum pratense* pollen versus pollutants-exposed pollen protein content (combination of gases or one by one fumigation), (ii) a decrease in some allergen recognition in some IgE binding proteins (whatever the gas used), and (iii) an acidification of several allergens on O_3_-exposed pollen and O_3_ + NO_2_-exposed pollen extracts [175]. The differences observed could be due to some posttranslational modifications at the protein level. With immunodetection experiments on pollen from the 3 Betulaceae species already cited [153], it was possible to show higher IgE recognition by patient sera sensitized to the pollen extracts from all NO_2_-exposed samples with comparison to the nonexposed ones. On* Dactylis glomerata* pollen again, when using nitrocellulose immunoprint technique after agarose isoelectric focusing separation, some other results did not allow detection of modification in the allergen isoelectric points after different experimental pollutant treatments (car exhaust gas, cigarette smoke, UV light, and ozone). However, some decrease in the extracted allergen amounts was noticed mainly after car exhaust gas and cigarette smoke exposure [149]. It can be noted at this step that DEP and tobacco smoke have many common characteristics, namely, particulate nature and shared chemicals [179]. Benzo(a)pyrene is an important component of DEP and causes abnormalities during pollen development of* Helianthus annuus* L. [180]. The different experimental conditions used to study the effects of pollution and the results obtained are summarized in Table 1 for birch pollen and in Table 2 for grass pollen.

**Figure 1:**
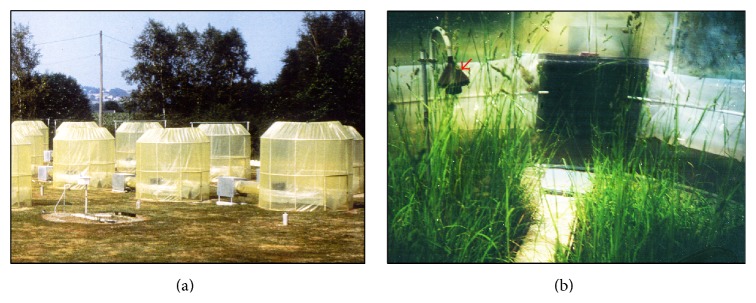
(a) Open-top chambers (OTCs) were located at Montardon site (10 km north of Pau, France). Technical characteristics of OTCs, close to those described by Heagle et al. [32, 33], have been already reported [34]. Each circular OTC had a diameter of 3 m and an open-top diameter of 2 m and was 2.8 m tall. It consisted of a galvanised iron frame covered with a polyethylene foil (Deltatex T2E). Ozone-free air (filtered air) or O_3_-enriched air was blown all around the chamber above the canopy level. The flow rate was controlled to achieve an air exchange rate of 3.14 times per min at the canopy level. When supplied, extra ozone was generated by electrical discharge of pure oxygen and injected into the air stream. Extra ozone was equally released only from 10 a.m. to 5 p.m. (GMT) in order to simulate the normal period of ozone exposure. The control chamber received filtered air (before ambient air was blown in the chamber, it passed through a charcoal filter that removed almost totally ozone), while, in O_3_-enriched OTC, O_3_ concentration reached 100 *μ*g/m^3^. Ozone was sequentially monitored in the three OTCs with an UV ozone analyser (Environnement SA, O_3_ 41 M) under the control of a computer recording system. (b) Inside a filtered air chamber: in the foreground mature* Dactylis glomerata* plants with inflorescences can be seen. The air suction device of the OTC allowing measurement of the ozone concentration is noted with a red arrow.

*(ii) Greenhouse Conditions*. During 2 consecutive seasons,* Dactylis glomerata* seedlings were exposed in open-top chambers designed as described by Biolley et al. [34] and supplied by filtered (Figures 1(a) and 1(b)) air or air containing O_3_ (100 *μ*g/m^3^, 8 hours per day) during 2 months preceding the pollen harvest.

**Figure 2:**
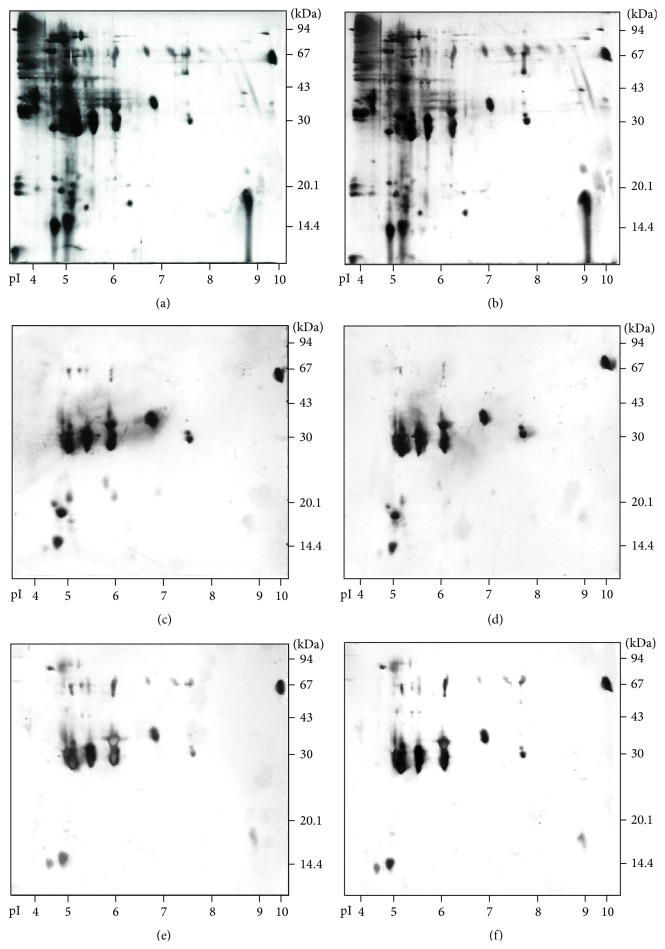
Two-dimensional gel electrophoresis analysis of the water-soluble proteins from* Dactylis glomerata* pollen harvested in ventilated greenhouse with air (a, c, e) or with air containing ozone, 100 *μ*g/m^3^, 8 hours per day (b, d, f). Pollen extract from* Dactylis glomerata* was submitted to an initial isoelectrofocusing separation followed by gel electrophoresis with SDS. The gels were either silver stained (a, b) or transferred on nitrocellulose incubated with 2 different grass pollen-sensitized patient sera (c–f). IgE binding was revealed using heavy chain specific antibody coupled to alkaline phosphatase (AP). The AP activity was detected using 5-bromo-4-chloro-3-indolyl phosphate and nitroblue tetrazolium (Sigma) in 0.1 M tris buffer pH 9.5. Isoelectric points (pI) values (at the bottom) and relative molecular mass (kDa, on the right) are indicated for each gel.

After pollen sampling, protein concentrations of the extracts [175] were measured. A slight but not statistically significant difference was observed in pollen protein concentrations from exposed (37 mg/mL) versus unexposed (33 mg/mL). But no difference at all was observed, by two-dimensional (2D) gel electrophoresis separation, in quality of these pollen proteins from plant material (Figures 2(a) and 2(b)). The allergenicity of the unexposed (control) and O_3_-exposed pollen was evaluated by 2D immunoblot electrophoresis analysis of the extracts (Figures 2(c)–2(f)). Using sera from 2 different grass pollen allergic patients, no significant difference was observed in either the IgE or IgG_4_ patterns [181]. In the same way, when comparing Amb a 1 content, as tested by ELISA, between 80 ppb O_3_-fumigated material versus 40 ppb O_3_-fumigated control one, no direct influence was detectable in Kanter et al.'s work [142].

*(3) Protein Nitration and ROS Formation*. Several lines of evidence indicate that most air pollutants can enter the plant tissues, as already mentioned, and act primarily through the production of ROS also called oxidative-free radicals [182]. Three important ROS, hydroxyl-free radical (neutral form of hydroxide ion OH^−^), superoxide anion (O_2_^−^), and hydrogen peroxide (H_2_O_2_), are highly toxic and can lead to damage of proteins, lipids, and DNA. For instance, ROS attack proteins to form carbonyls and can react with nitrogen species then to form nitrotyrosine with tyrosine and with lipids to generate ethane and isoprostanes. These reactions could have an impact on membrane organization. ROS also can react with DNA to form base pair adducts, such as 8-oxo-2-deoxyguanosine affecting its structure [183, 184]. They are clearly suspected to play a major role in allergic inflammation [185]. In the same time, laboratory experiments show that proteins are efficiently nitrated upon exposure to gas mixtures of NO_2_ and O_3_ or one ofthe different mixes composing polluted urban air. The nitration reaction leads to the addition of nitro groups to the aromatic rings of tyrosine residues in the polypeptide chain, and this posttranslational modification can enhance the allergic potential of various proteins [173, 174, 186]. Apart from the chemical ageing of some air PM, ROS may also participate in the formation and growth of multifunctional organic substances [187]. It is also known that quinones present in PM may exceed those of both ROS and free radicals [188]. Experimentally, Pasqualini et al. could show that O_3_-exposed* Ambrosia artemisiifolia* pollen can become more allergenic through stimulation of inflammatory ROS-generating NAD(P)H oxidase [157]. Even if Bacsi's group already proved through several very precise studies on ragweed pollen and pollen from more than 30 other plants that unpolluted material (both commercial Greer Laboratories' one and collected whole grains as well as subpollen particles) can generate ROS-producing NAD(P)H oxidases [121, 189] and if, nevertheless, Shalaby et al. showed that intrinsic NAD(P)H oxidase activity (or other enzymatic activity) is not a universal mechanism conferring allergenicity to pollen [190], it seems clear that an exacerbated activation of these phenomena is possible through urban pollutants.

#### 2.2.3. Subpollen Particles, Pollen Fragments, and Free Allergens

Experimentation on polluted pollen-derived material was developed on different aspects. It concerns, till now, the controlled pollution of innate inner subparticles (considered as “pollen cytoplasmic granules,” PCG) of* Phleum pratense* grass pollen, the fixation of free allergens on DEP, and the release of fine particles from several pollen sources following impaction.

The experimental pollution by NO_2_ and O_3_ at various rates (resp., from 0.5 to 50 ppm and from 0.1 to 5 ppm) of* Phleum pratense* pollen leads to fragilization of the exine and increasing of the released amount of inner material. The most significant effect of NO_2_ was between 2 and 50 ppm when O_3_ exposure plays a role even at the lower 0.1 ppm level. It was thus possible to conclude that damage of pollen grains by traffic-related air pollutants such as NO_2_ and O_3_ can lead to spontaneous release of inner subparticles from different grass pollen sources [145, 191, 192]. Using immunogold labeling techniques, Knox et al. could visualize the* in vitro* binding between diesel exhaust carbon particles (DECP), positively charged (30 to 60 nm), and Lol p 1, the major grass pollen allergen from* Lolium perenne*, when Lol p 5 showed essentially no binding. As an acidic glycoprotein, Lol p 1 may bind to these particles because of its negative charge (at pH 6.8) [193]. In the same way, Ormstad could show the fixation Bet v 1 on DEP [194]. These authors consider that if Bet v 1 can bind at these particles, other proteins could do so as well. From this point of view, the binding could be pI-dependent (negative charges promoting the binding) or related to glycosylation degree of the allergen. As Knox et al., in previously cited paper, they proposed that the role of the negative charge of the allergen is of major importance for the binding with DEP.

**Figure 3:**
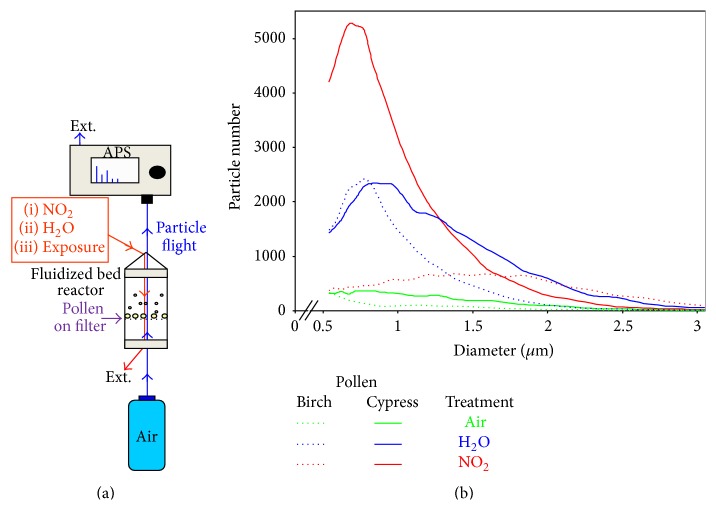
Experimental exposure of cypress and birch pollen with nitrogen dioxide (NO_2_). (a) Cypress (25–30 *μ*m) or birch (20 *μ*m) pollen grains, filed on filter (0.22 *μ*m), were aerosolized into a synthetic industrial air flow of 100 mL/min (allowing the flight of smaller particles than pollen) and sent into an impactor equipped solely with a PM_10_ stage. Particle size distributions were measured, during 10 min, at the outlet of the impactor with an aerosol particle sizer (APS) (0.5–20 *μ*m). (b) Pollen samples were moistened and then dried with air during 10 min (blue curves) and control samples were realized with industrial air (green curves). Impaction tests were also done with pollen grains artificially polluted with NO_2_ (0.5% for 10 min) (red curves). Particle size distributions were normalized to show an equal number of pollen grains at 17 *μ*m.

Experiments on aerosolized birch and cypress pollen grains, in experimental system, under various conditions (Figure 3(a)) showed that the emission of heterogeneous small particles increased when the pollen source was humidified (Figure 3(b), blue lines). This is likely the result of the release of subfragments and/or internal granules. Interestingly, while no effect of the exposure to NO_2_ (0.5% during 10 min) was observed on birch pollen (Figure 3(b), red dotted line) it could induce a 10 times increased emission of number of particles smaller than 500 nm for* Cupressus sempervirens* pollen (red continuous line). These submicronic particles likely corresponded to orbicules which are located on the outer surface of the pollen and are a hallmark of cypress pollen grains. This experiment strongly suggests that NO_2_ is able to strip off orbicules from pollen grains and thus release them as free subparticles in the atmosphere [195]. Inner subparticles from birch pollen grains were also shown to be released upon impaction on a solid surface at wind speed of about 3 m/s [196, 197].

## 3. Effects of Polluted Pollen Grains, Subparticles, and Derived Atmospheric Allergens on Model Animals, Allergic Patients, and Cell Material

### 3.1. Animal Models

The animal models could help understand the complicated links between atmospheric pollen material and main pollutants. Several series of work tried to mimic, on animals, what could happen for pollen allergic patients. When taking into account such studies, it is nevertheless important to keep in mind the existence of many biases regarding animals' choice [198–200] and/or protocols with, for instance, the dominance of ovalbumin-BALB/c mouse model [201, 202]. A first example, among many potential others, can be given: the extent of bronchus-associated lymphoid tissue (BALT) differs greatly among animal species. It has to be noted, at this step, that humans and nonhuman primates have little BALT. It probably plays a major role in development of allergic responsiveness. Another example clearly shows some of the great physiological differences: mice are obligate nose breathers, incapable of mouth breathing. The oral breathing in humans bypasses the effective air cleaning capacity of the nose [203]. Thus, model animal should always be appreciated only as surrogates [204]. Be that as it may, different trials were proposed, year after year, on mice, rats, and guinea pigs for either gases or PM which clarify some pollution-allergenic material links. With a recent review on animal model studies being available, we insist here on papers and works that were unlisted in it [205].

#### 3.1.1. Gases

When comparing results from IgE immune response experiments on mice that were injected with extract obtained from pollen harvested of* Dactylis glomerata* with exposed seedlings (O_3_ at 80 *μ*g/m^3^) and a pollen extract from unexposed plants, Charpin et al. did not find significant IgE rates differences in the 2 groups of mice [181]. In a work on recombinant allergen rBet v 1 from* Betula* pollen source nitrated by reaction with tetranitromethane dissolved in methanol, the Austrian-German team of Gruijhuijsen showed that levels of IgE, IgG_1_, and IgG_2a_ were higher in animals sensitized with nitro-(3,4)-Bet v than in unsensitized ones [154].

#### 3.1.2. PM and DEP Compounds

In a series of works by Fernvik et al., a set of mice (from strain selected for expressing intensively different sequalae of asthma after allergenic immunization and provocation) were immunized with (i) birch pollen (100 *μ*g/mouse) and either (ii) global tunnel dust (collected in Prague, Czech Republic) or (iii) one of the 8 purified different fractions from this traffic PM material (TPM). These fractions try to mimic the various chemical compounds adsorbed on TPM aerosols. Selected mice were provoked intranasally with either a mixture of pollen and TPM, a mixture of pollen and one of the 8 fractions, or, finally, pollen alone before a challenge with methacholine. The bronchial hyperresponsiveness (BHR), specific IgE-levels, and number of recruited eosinophils in bronchoalveolar lavage (BAL) were increased in mice immunized and provoked with the mixture of pollen and TPM. However, mice immunized with pollen only and provoked intranasally with pollen or a mixture of pollen and TPM showed higher levels of IL4 and IL5 [206, 207]. Results obtained with 8 different fractions showed the highest titers of IgE and BHR in the positive control mice (immunized and provoked with a mixture of pollen and TPM), followed by mice immunized with pollen and fraction 2 (which contains organic acids). They demonstrated also that fractions 2 (organic acids) and 7 (highly polar compounds) seem to contain potential adjuvants stimulating IL-5 production, the IgE synthesis, the eosinophil recruitment, and the BHR [207, 208].

When working on DEP pollutant role from Kanto (Japan) region, Maejima et al. observed a time-dependent increase in Cry j 1 and Cry j 2 allergens specific IgE ELISA titer in sera from mice exposed only to* Cryptomeria* pollen. On groups of mice also exposed to either filtered or unfiltered DEP, through nose instillation, these authors observed larger increase of the same type of signal. The study thus suggested not only that fine particles may enhance the production of IgE in mice exposed to pollen but also at the same time that the nature of the particles could be of importance, gas components of DEP, namely, as adjuvant factor regarding IgE production [209, 210]. The same kind of results was observed with mice for which* Cryptomeria* pollen extract was intraperitoneally delivered. A persistent IgE response in pollen and DEP immunized animals was detected, while it was not the case with only pollen immunized mice [211]. An increase of IgE level against* Cryptomeria* pollen associated with DEP was also shown on not yet (fetal stage) and newly born rats during differentiation of their immune system [212]. This last work showed the very important role of ultrafine PM in the elevation of IgE against pollen extract. On herb pollen material, the number of pollution-pollen links' studies is rather less than that on trees ones.* Phleum pratense* pollen grains induce an allergic response in Brown Norway rats after intranasal and intratracheal administrations. On animals challenged at day 21 (pollen 10 mg/mL, DEP 3 mg/mL), DEP has an adjuvant activity on the IgE production [213]. But, in a later work, the same Dutch team showed that, in the BAL of rats from the same strain exposed to pollen-PM mixture, the percentage of eosinophilic granulocytes was lower than the one of rats only exposed to pollen [214]. Studying also the allergic response in Brown Norway rats, Rogerieux et al., when comparing* Phleum pratense* pollen sensitization, did not find any significant difference between controls (saline injected) versus raw and filtered DEP exposed animals. There was no pollen specific humoral allergic response (specific serum IgE). There was no modification of the intensity of the pollen-induced cellular activation and eosinophil influx. The only one response, assessed by alveolar macrophages infiltration in lungs, was inflammation detected in rats exposed to filtered DEP [215].

Among DEP compounds, the potential role of benzopyrene and 1-nitropyrene in allergic rhinitis aggravation was evaluated by Nabe and Mizutani in guinea pigs [205]. In this animal model, these components did not seem to affect the IgE-dependent activation of mast cells. Nevertheless, from a study using intranasally immunized mice, it sounds clear that the adjuvancy of polycyclic aromatic hydrocarbons (PAHs) in DEP may be of importance in the production of IgE against* Cryptomeria japonica* allergens (namely, and mainly, Cry j 1). Indeed the IgE responses in mice immunized with (i)* Cryptomeria* pollen extract and pyrene, (ii) the same pollen source and total DEP, or (iii) pollen source with anthracene, fluoranthene, and benzopyrene were significantly enhanced compared with experiments with only pollen-immunized animals. Furthermore, when incubating intraperitoneal macrophages obtained from the unimmunized control mice with pyrene, anthracene, fluoranthene, or benzopyrene, IL-1 alpha production of the macrophages was observed in each case [216].

A last example showed that, on a very specific animal model, mice with severe combined immunodeficiency transplanted with human peripheral blood lymphocytes (*n* = 39) and thus producing human IgE, after early stimulation with birch pollen (batch pollen suspension at 2 mg/mL), even if a tendency to higher total IgE levels was observed, after DEP treatment (10 mg/mL), no statistically significant effect of this pollutant could be detected [217].

#### 3.1.3. Combined Gaseous and Particulate Pollutants

Using a guinea pig model, Rezanejad and Madj proposed results on polluted* Lagerstroemia indica* pollen material versus unpolluted ones [218]. They found no significant IgE level change between unpolluted and polluted pollen immunized animals. When comparing* Pinus eldarica* pollen material exposed in polluted Tehran zones versus fresh gathered unpolluted pollen, the same team found higher eosinophilia (14% more) for guinea pigs injected with polluted material [219]. On pollen from herbs, results of Madj's team on* Tagetes patula* and* Spartium junceum* pollen material showed higher values of eosinophils, neutrophils, and IgE for animals injected with extracts prepared from polluted pollen [218]. It was also the case in the work by Arbabian and Entezarei on* Triticum aestivum* [220]. In a study on* Canna indica* pollen, using BALB/c mice, Madj et al. found that the allergenic potential of the polluted chosen material is higher than the nonpolluted one. Through their different investigations, they showed that the skin tests wheals were larger with polluted material (mean diameter: 3.1 versus 5.6 cm) and the eosinophil as well as the neutrophil number was higher (resp., 39 versus 58 and 84 versus 93 × 10^4^ cells/mL of blood). The IgE level was also increased (7.8 versus 12 ng/mL) and regarding the IgE-specific immunoblotting, no significant difference was detected between the 2 groups [75].

### 3.2. Humans

Although investigations on the effects of pollution on health are numerous, studies on allergic or healthy human individuals are fewer than those on animal models [179]. Surprisingly, in some of these published papers the contribution of pollen as bioaerosol is omitted [6, 221–223]. Nevertheless,* in vivo* as well as* in vitro* studies are available which take into account (i) the human mucosal system, (ii) the 100–140 m^2^ of human lungs, (iii) the interactions between aerosols and mucosa through the daily 10,000–15,000 liters of air entering an adult, and (iv) the molecular and cellular immune parameters involved in allergic diseases (IgE, cytokines, eosinophil, neutrophil, basophil, mast cells, T and B cells, etc.).

#### 3.2.1. Experimental* In Vivo* Allergy

*(1) Gases*. A Swedish team showed in a series of sophisticated works (1997–2005) on grass and birch pollen allergic and asthmatic people experimentally submitted to NO_2_ in exposure chamber that (i) for grass pollen allergic patients (*n* = 18) a 30-minute exposure to NO_2_ (490 *μ*g/m^3^) gave a higher bronchialresponse to the allergen, mainly during the late phase response [224], (ii) for grass (*n* = 4) and birch (*n* = 12) pollen patients repeated exposure for 4 days to 500 *μ*g/m^3^ for 30 minutes prior to a nonsymptomatic allergen dose enhanced not only the early but also the late phase airway response [225], (iii) with the same kind of patients (*n* = 13) NO_2_ + allergen exposure enhanced the percentage of neutrophils in both bronchial wash and BAL versus air + allergen one with levels of eosinophil cationic protein in bronchial wash higher for the NO_2_ + allergen group and no effect on pulmonary function [226], and finally, (iv) regarding again inflammatory reactions ambient levels of NO_2_ can prime circulating eosinophils and enhance eosinophilicactivity in sputum in response to inhaled allergen [227]. On NO_2_, another study by Wang et al. allowed showing that NO_2_ can have effect on eosinophils. The experimental work was done on 16 subjects with a history of seasonal rhinitis but without other symptoms. They were exposed to either air or NO_2_ (6 h at 400 ppb) and challenged or not with commercial mixed grass pollen extracts. While no changes in the levels of eosinophil cationic protein, mast cell tryptase, and myeloperoxidase (from nasal lavages) were detected for the tested people without challenge, it was possible to observe some modifications notably in the markers of activation of eosinophils for the allergen-challenged subjects [228].

The idea that links between ozone and allergic people exist occurred very long time ago, for instance, explicitly, in Blackley's mind. Moreover, when writing the D section of his famous book [229], he suggested that variable levels of atmospheric ozone might variably affect allergic disorders (hay fever). Regarding the first experimental measures linking O_3_ and allergy, they were made, of course, from mid-20th century mainly in the perspective of man-made O_3_ emission changes through industrial and urban life activities.

On O_3_ effects, Molfino et al. could prove that, even at low concentrations (“similar to those found in large urban cities,” i.e., among the largest 1990s towns in the USA and Canada) in 10 tested atopic patients' set (positive skin responses to grass or* Ambrosia* pollen), an increase in the bronchia responsiveness to allergens (subjects challenged with methacholine) was detectable without affecting baseline pulmonary function. After O_3_ exposure (0.12 ppm) for 1 h at rest, the dose of inhaled allergen necessary to elicit the same early allergic response was half that for allergen preceded by air inhalation (control) [230]. Nevertheless, according to differences in experimental conditions (larger O_3_ exposure chamber, e.g.), controversial results were also produced. Hanania et al., notably, on 15 subjects challenged with either grass (*n* = 9) or* Ambrosia* (*n* = 6) pollen extracts, could show that, at low O_3_ level, there was no significant effect of the gas on airway allergen responsiveness [231].

On a work on SO_2_ fumigated,* Argemone mexicana*, pollen, Parui et al. found, through skin prick test analysis on 43 patients (of which 44% were sensitive to this specific pollen), an increase in the number of patients showing positive response to the experimentally polluted pollen of this plant. Respectively, 46%, 52%, and 54% of the patients were found to be sensitive to the pollen extracts of 24-, 48-, and 72-hour fumigated pollen [168]. In the same way, Huss-Marp et al. investigated the effect of volatile organic compounds (VOCs: mixture of toluene/m-xylene) and SO_2_ on grass pollen (*Phleum pratense* L.) allergenicity as measured* in vivo* by skin prick tests (SPTs) in patients with grass pollen allergy. Pollen exposed to VOCs released elevated levels of Phl p 5 and PALMs and led to an enhanced SPT reactivity. No such effect was seen for SO_2_ pollen exposure [177].

*(2) PM and DEP Compounds*. The team around Diaz-Sanchez from UCLA School of Medicine (Los Angeles) proposed in the 1990s different works focusing on the links between* Ambrosia* pollen allergen (Amb a 1) and DEP on human subjects. Regarding the interaction of DEP on allergen-driven responses* in vivo*, this team showed that DEP enhanced the allergic specific IgE response but not the total IgE response in ragweed-challenged subjects sensitive to pollen (*n* = 13). Amb a 1 specific IgE was 16 times higher following the challenge with* Ambrosia* pollen allergen associated with DEP compared with challenge with Amb a 1 alone. Regarding the detected alteration in cytokine mRNAs (collected from cellular RNA recovered from nasal washes through reverse transcription-PCR), it was also clear that Amb a 1 allergen associated with DEP enhanced the absolute levels and altered the relative levels of *ε* mRNA isoforms. It can be assumed, following Diaz-Sanchez et al., that Amb a 1 allergen provides the orientation towards the Th2-like immune pattern and that DEP amplifies this response. Allergen exposure may result in an early release of IL-4 which is thought to be critical to the development of Th2 responses and can inhibit the development of Th1-type effector cells [232]. On a later study, the results obtained from the analysis of 8 experimentally treated patients indicated that the combination of mucosal stimulation with DEP and* Ambrosia* pollen allergenic source is capable of driving* in vivo* isotype switching to IgE in allergic patients sensitive to* Ambrosia* pollen. Such switch could mean that B cells initially expressing IgM and/or IgD on their surface rearrange the active encoding variable-diversity joint region to other Ig heavy chain loci and thereby could provide antibodies with different effector functions but the same antigen activity [233]. All these results tend to prove that increasing DEP (mainly linked to higher road traffic emissions) with unchanged levels of allergen could be one of the factors in the observed increasing clinical sensitization and prevalence of allergic respiratory diseases.

The protocol proposed by the group of the Harvard School of Public Health (Boston) used residual oil fly ash (ROFA) as pollutant and aerosolized whole pollen grains (from ragweed, red oak, or white birch) according to the differential positivity of the SPTs of the atopic (*n* = 5) and nonatopic (*n* = 3) subjects in an experiment based on 3 exposure challenges [234]. The people involved were submitted to (i) ROFA without pollen material, (ii) clean air + pollen material, and (iii) ROFA + pollen material. Such work provided evidence of a greater-than-additive interaction between ROFA exposure and pollen challenge. A 1 h ROFA exposure, 3 h before pollen challenge, enhanced the nasal inflammatory response. It consisted of an increase in total leukocytes, neutrophils, macrophages (cells from nasal lavages), and interleukins, IL-4 and IL-8. It can be noted that atopics had enhanced IL-4 and increased inflammatory response. It was not the case for nonatopic patients, who had an enhanced IL-8 response like the 5 atopic ones.

A UK team from Birmingham, around W.S. Tunnicliffe, experimented on particulate sulfates. This PM fraction is most of the time the result of an atmospheric oxidation of SO_2_ to sulphuric acid (H_2_SO_4_). H_2_SO_4_ exists in the air in particulate form. It reacts with NH_3_ to form either NH_4_HSO_4_ or (NH_4_)_2_SO_4_. The 13 atopic subjects were submitted to PM-H_2_SO_4_ (at 100 *μ*g/m^3^ or 1,000 *μ*g/m^3^) and challenged with* Dactylis* and* Phleum* pollen material. These results suggest that, at least at the highest main concentration, fine PM-H_2_SO_4_ can potentiate the early asthmatic response of asthma patients to inhaled grass pollen allergens [235].

*(3) Combined Gaseous and Particulate Pollutants*. When the already cited Swedish team studied the effects of a city (Stockholm) road tunnel air pollutants (gas, PM_10_ and PM_2.5_) for a total of 20 patients (grass (*n* = 7) and birch (*n* = 13)), they found that a 30-minute exposure (in a car during the rush hour) enhanced the asthmatic response to allergen (from freeze-dried birch or timothy grass pollen extracts) inhaled several hours later, although pulmonary function was not affected [236].

In an experiment showing that the release of Phl p 5 was only lowly influenced by the interaction with PM from “road dust” (4 hours in fluidized bed reactor on artificially polluted* Phleum pratense* pollen), it has been shown that, nevertheless, the water extract (from the same polluted pollen source) used for producing skin prick test could give enhanced reactivity (30–45%) for a set of allergic patients [136].

#### 3.2.2. Experimental* In Vitro* Allergy with Human Cells

Several works on cellular material showed different effects of pollution on allergenicity through pollen material. For instance, by measuring neutrophil migration in a way close to what was proposed in the 1970s and 1980s [237–239], Traidl-Hoffmann's team evaluated the chemotactic activity of rural versus urban birch pollen aqueous extracts. Using neutrophils from 11 nonatopic donors, they found, from this experiment, that pollen material from urban areas exhibits a significantly higher chemotactic activity compared to pollen from rural areas [96]. A neutrophil migration experiment was also developed with pollen from differentially O_3_-exposed birch trees (85 *μ*g/m^3^ versus 54 *μ*g/m^3^), showing highest chemotactic activity scores with the most exposed material [98].

On basophils from 6 atopic birch pollen allergic patients and 5 healthy persons used as control, people from the same Munich ZAUM center associated with other German researchers could evaluate the role of PM aerosol on these specific blood cells [240]. Working with a commercial BASOTEST kit for their basophilic activation test (BAT) in a flow cytometry experiment, they found that incubated with DEP (PM_2.5_) and rBet v 1 (from* Betula* pollen source) basophils versus incubated with rBet v 1 alone ones expressed significantly more CD63 proteins on their surface membrane. With basophils being known to represent a major source of early IL-4 cytokines production in allergic patients, such cellular results were of real interest.

On dendritic cells from peripheral blood monocytes, recent results from the already cited study by Beck et al. showed that extracts prepared from pollen with low O_3_ exposure had more effect (inhibiting the IL-12 response) than pollen with high O_3_ exposure. But a role of nonallergenic adjuvant PALMs has to be taken into account [98].

A fourth kind of cells can be cited here as an example of the different* in vitro* studies proposed year after year: respiratory epithelial ones. Again* Betula* pollen was at the center of the experiment trying to evaluate the role of Pb, one of the major current MTE, in pollen allergenicity. The protocol was carried out with 2 groups of pollen (experimentally polluted versus unpolluted) and, within the polluted ones, the pollutant dose was either 30 mg/L or 60 mg/L. The results on incubated cells show a dose- and time-dependent increase in IL-5 (“allergic” cytokine) gene expression (mRNA levels) on Pb-exposed birch pollen material [241].

## 4. Combined Effects of Atmospheric Pollution and Biological Material Produced from Pollen Grains on Allergic Population

As already mentioned, a high number of studies are available regarding the direct pollutions effects on health [242]. We thus should here only insist on the fact that allergic people, without being in contact with polluted pollen and/or bioaerosols issued from polluted pollen material, can be fragilized by pollution* largo sensu* itself. It can happen before and/or after the so-called pollen season when the bioaerosols from pollen are fewer (around 2-3 months per year: from mid-autumn to mid-winter). It can also happen within the different phases of pollen release, from January (some Betulaceae and Cupressaceae) to late October (with Compositae like* Ambrosia* in many parts of the temperate regions) [243]. Taking the question from another angle, it is obvious that the different kinds of pollutants often do not occur during the same period of the year in high-density human zones. The example of the summertime O_3_ pollutant is clear. From its precursor NO_2_, O_3_ mainly demonstrates its health-degrading effects at low atmosphere levels under solar radiation. Thus, its effects on allergic people are more likely to be associated with the presence of deep atmospheric pollen charge. In experimental conditions on guinea pigs and healthy subjects, the effect of O_3_, used alone, at relatively low concentrations, was evaluated on bronchial epithelial cells and alveolar macrophages that produce different proinflammatory mediators [244]. For instance, 30-minute O_3_ exposure at 200 *μ*g/m^3^ increased TNF*α* secretion as well as IL-6 and IL-8 levels by the alveolar macrophages. In general, O_3_ is a potent oxidant that produces free radical and ROS. The epithelial surface of the respiratory tract is particularly rich in oxidants such as glutathione and ascorbate [245].

But at the same time, apart from model animals and human health ones, its other bioeffects, also ROS-generating in many plants [246], are not necessarily on aerosols from all kinds of airborne pollen material and/or, for instance, spring-pollinating Gymnosperm and Angiosperm species in and outside main population concentration zones. Of course_,_ interactions with other pollutants (namely, DEP) [247] are quite frequent, even if not occurring in all circumstances. For real, O_3_ “can potentiate the airway response to inhaled allergens” [248].

Pollutants have on epithelia, acting usually as physicochemical barriers at nasal and bronchial levels, potential morphological and functional effects inducing notable changes on the tissues. With the mucociliary clearance being defective through pollutants action, firstly the allergens can stay on epithelial surface and secondly the diffusion of proteins in the subepithelial cell-abundant layer increases. Thus, in such a way, allergens can access more easily immune system's cells. This alteration of the defense barriers by pollutants can be gas-induced and/or particulate matter-induced. For instance, human lung parenchyma retains PM_2.5_ while particles larger than 5 *μ*m and <10 *μ*m only reach the proximal airways where they are eliminated by mucociliary clearance if the airway mucosa is intact. Pollution alone can therefore play an undoubted role in the amplification of the response (at nasal and bronchial levels) to inhaled pollen allergens. Furthermore, it has been shown that even on healthy people the nasal instillation of DEP at realistic concentrations induces a dose-dependent increase of IgE in the nasal-wash liquid. Diaz-Sanchez's team experimented on the exacerbated IgE response* in vivo* in the human upper respiratory tract [249] and proposed that pollutants could enhance on-going IgE production directly by acting on B cells [179]. On their side, Casillas and Nel [250] have suggested that DEP may act as adjuvant in a manner similar to Al(HO)_3_ in promoting a Th2-type immune response. DEP are powerful contributors to nasal inflammation and hyperresponsiveness [251].

In consequence, it is important to keep in mind that pollutants by themselves could be “pollen allergy-initiating” and/or “pollen allergy-facilitating.” If, at least epidemiologically, attested links between air pollution and airborne pollen charge are still controversial [252, 253], nevertheless combined effects of atmospheric pollutants and polluted bioaerosols issued from pollen material seem, from experimental works, to be, potentially, highly unfavorable to allergic people.

Allergenicity is a complex process, like immunogenicity, because it includes several characteristics of, first, the triggering compound, such as its dose, its route of entry, its frequency of administration, and the physicochemical and functional nature, and, second, several characteristics of the host such as the age of sensitization and the general current health of the individual. An efficient immune response relies on the stimulation of the innate and acquired immunity. Innate immunity involves cells and molecules immediately available and their activation through pattern recognition receptors results in a first barrier defense of inflammatory type which is also a triggering signal for acquired immune response, more specific [254]. Pollutants, gaseous or particulate matter, can be considered as injury signals and, as such, commit cells and molecules of the innate immune system: epithelial cells, macrophages, and molecules coating mucosal tissues. In consequence pollutants, by enhancing the formation of ROS and inducing inflammatory responses, behave as adjuvants reinforcing in some conditions the allergenic power of the allergens by activating Th2-driven acquired immune response.

From the hygiene hypothesis proposed 20 years ago advocating the importance of microbial stimulation for an antiallergic Th1 immune response, it was shown that, more than amount of stimulation, a diversified stimulation was required to ensure an unbiased immune system, properly educated [255–257]. The microbiota, that is, the collection of bacteria colonizing an individual, is now considered as the biosensor of the organism. An unbalanced biodiversity in commensal bacteria reflects or is reflected by a disease. In consequence, any environmental factor that can modify the microbiota may induce a pathological situation. Among these factors are the westernized diet, antibiotics, and delivery modes [258]. The potential effects of pollutants on microbiota require more investigations.

In summary, pollutants can induce structural modifications on allergen molecules themselves. However, the effects are moderate on intrinsic allergenicity but rather enhance the susceptibility of the host inflammatory and immune responses through mechanisms involving overstimulation of innate immunity and impairment of immunoregulatory circuits such as those controlled by the microbiota. Furthermore, several studies showed that epigenetic mechanisms could be involved in increased allergic disorders observed in westernized countries [259].

## 5. Methodological Biases and Proposed Research Tracks

### 5.1. About Biases

#### 5.1.1. Controllable Biases (Some Examples)

*(1) Pollen Material and Source*. The pollen material used for studying the links between pollen and pollution is, in most of the cases, precisely described in the produced papers. But, in some others, it is still not possible to find indications about how the pollen was collected and even from which “polluted” (not clearly defined) regions it was gathered [220]. In some experimental pollution works, the source of the material stays unknown: “mature anthers of* Cocos nucifera* and* Datura metel* were collected separately” [167].

If in many cases material is collected in several points of a clearly defined polluted zone, in some other publications, the source sounds unique (at least no indication is given). In some of the numerous papers on polluted birch pollen material, there was no mention of the studied species on which experimental work was done [66], when it is known, like for many other plants, that, for this tree, interspecies differences exist in terms of pollen protein and allergen content, for instance.

*(2) Pollutants*. The doses and types of atmospheric pollutants used in experimental pollution are diverse, but are they always realistic and allowing comparisons?

One of the key points discussed by Behrendt's group in the 1990s was about 2 different types of pollution, East and West Germany ones, just before and after the reunification. SO_2_ pollution model was, at European scale, the old one, generating less allergic diseases, when the high road traffic-related western pollution could have explained more easily the increase in allergy. This point of view sounds well grounded even if some recent works tend to show that lifestyle changes (other than higher cars availability and traffic) were also important in order to explain the West-East new allergy trends in reunified German regions [260]. Nevertheless, if O_3_ and PM are now some prevalent air pollutants worldwide, the coal-derived pollution still exists in many regions of the world and is associated with always higher and higher traffic emissions [261]. Then, when experimenting, it should be of importance to select, according to the local conditions and plants present in the environment, the most pertinent pollutants to be tested at doses that sound credible on specific pollen material. For instance, Asia is not Europe and half of the world population lives in Asia. In contrast to the relatively homogeneous western countries, the environment in Asia is very diverse and there are tremendous variations from one region to another in terms of either genetic background or atmospheric pollution load [261–264].

In Europe, a list of 12 air components which pose a health hazard has been proposed by the European Union's administration: NO_2_, SO_2_, O_3_, CO, C_6_H_6_, PM_10_ (in PM_10_: Pb, As, Cd, Ni, and benzopyrene), and PM_2.5_. And, for instance, unfortunately, no works on As- or Ni-exposed pollen seem available, there are few on benzopyrene [180], and a single recent study is noticeable on effect of cadmium on* Picea wilsonii* pollen germination [265]. As for the pollen material and source, it seems necessary to always indicate the levels and concentration values of pollutants used in experiments.

*(3) Animals*. As mentioned previously, considerable differences exist among models regarding, for instance, the following aspects: the animal species used (only rats, mice, and guinea pigs were taken into account here), the route of antigen administration, the protocol for both induction and elicitation of responses, the type of response measured, and also the criteria for some conclusion regarding ≪significant≫ reactions. Examples abound where adverse effects to a particular substance were noted in certain species, and in particular organs of the species, but the same effect was never observed in humans [204]. Nevertheless, even if it seems clear that cell studies should be promoted, works on animals are of importance if comparisons are possible with reproducible protocols on a reasonable number of individuals.

As for other experimental parameters, precise notations are needed in order to evaluate the relevance of works on animals. It is not always the case [220].

#### 5.1.2. Uncontrollable Biases (a Short List)

*(1) Life and Death of Pollen Grains*. Pollen grains released in the atmosphere are equal neither in maturity nor in velocity. When anthers from tree catkins or ground level plants open, what is disseminated is a mixture of grains that are not fully at the same biochemical maturation stage. The aerosol issued from this plant dust can differentially stay alive or not for some hours according to global air conditions (i.e., physical ones like temperature, moisture, electricity, and wind and chemical ones including humans generated pollution). Apart from these first considerations, one can think of fragments discarded, from the initial stage, everywhere later on [112, 266, 267], as PM components [268] or even as parts of some more global organic carbon [40], but with, again at this level, various protein conditions (washed for some of them, still resistant to atmospheric aggressions for some others). It is quite complex to mimic* in situ* conditions even with fine methodology as the one proposed recently by Beck et al. [98] or Buters et al. [269]. Tracking precisely either hydration levels or maturity stages for plants growing in various conditions [43, 160], even in a not very large zone of study, stays, at least for the moment, not under full research control. 

*(2) From Where Do These Polluted Bioaerosols Come?* When speaking of polluted bioaerosols, it is often assumed that this biogenic fraction was atmospherically polluted. Nevertheless, it is, in most of the cases, not possible to prove it, and in the same time, from soils [55, 270] to growing plants, it is obvious that pollution can interfere in anthers and pollen grains formation [271] before maturity. It has been proven that low but real Cd, Ni, and Pb concentrations exist in pollen measured simultaneously with polluted soils [272]. On the one hand, from the atmospheric water content, washed pollen grains could be deprived of soluble allergens and lixiviated plant fragments; also, aerosolized allergenic material could be dispersed. And, from the experimental protocols themselves, what is considered as polluted pollen material to be studied could have been polluted on the impactor itself during the sampling phase.

Questionable elements are also linked with the fact that it is of course known that terpene hydrocarbons that emanate from plants as well as natural low molecular weight olefins are chemically quite close to automobile emission particulate matter [273].

*(3) Long Life of Pollution Contributors and Atmospheric Dissemination of Pollutants*. Many pollutants are quite resistant and persistent in the environment. The adverse effects of some MTE (e.g., Pb, Zn, Ca, and Cu) on the plants and then on the airborne pollen grains as well as aerosolized pollen material issued from them may not be immediate but delayed by several days, months, or years [78] and what about the potential effects and pollen damage due to herbicides, pesticides, and weed-killer agents [65, 274, 275] on allergic people?

### 5.2. Five Potential Research Tracks for Studying “*Polluen*” and Its Effects

When trying to synthesize what has been proposed in terms of studies on pollution-pollen links at different levels, from plants to humans and their diseases, one can also see what has not been experimented or poorly experimented. It seems quite relevant to propose, at least, some tracks to explore in a deeper way the so special and still enigmatic links between pollen and atmospheric pollutants.

But before doing so we need to give some precision on what we want to talk about.

Because as shown for long quite clearly by Behrendt and the successive research teams, notably in her 1997s paper [276], (i) polluted particles are carriers of not only pollutants, gaseous and/or particulate, but also allergens and (ii) atmospheric pollen material is carrier of not only allergens but also multiple pollutants, we proposed to put forward the neologism “*polluen*,” previously used by some authors, such as Peltre and Laaidi et al. [21, 65], in order to point this specific atmospheric material out. Such aerosol, in many cases, between organic and inorganic, between biologic and amorphous, and between liquid and solid aerosol, could be considered as an alien material or even as some xenobiotic. It takes many forms (e.g., containing either still water-soluble allergens or just non-water-soluble ones) and many shapes and sizes (from submicronic able to interact with lung tissues to millimetric and stoppable by nasal epithelial cells) and it has different properties (notably, changes in allergenicity, cytotoxicity, or adjuvanticity). When using this generic* polluen* word, we also would like to insist on the fact that the conditions for studying such material should really be specific and are difficult to be assimilated to the only ones of pollen or pollution or even aerosol studies. When taking such point of view, such pragmatic approach, it thus interacts with the diverse experimental devices and protocols to be promoted.

*(1) Flow Cytometry Approach: From Pollen Grains to Human Cells without Forgetting Subparticles*. Flow cytometry is a powerful technique, massively used not only in the biomedical field for years but also in the plant sciences' one [277]. It is now considered as a potentially efficient tool for the evaluation of atmospheric pollen and fungi allergenic load [278, 279]. If valuable for collected outdoor ambient material, it can be of interest also in indoor experimental studies. Several very worthy studies already opened this way, in the* polluen* field, exploring, for instance, the adjuvant effects of DEP on activation parameters of human basophils from birch allergic patients' blood as already mentioned [240] or, even more precisely, on different DEP-PAHs in the same experimental context [280, 281]. The work by Verstraelen et al. [282] on dendritic cells exposed to various DEP concentrations, in the presence or absence of lipopolysaccharide as activating source, gives the idea to try some experimental study in the same direction, with several allergens, in order to understand, firstly, the changes in the surface expression of the most relevant CD molecules listed as cell surface markers of allergy, allergic inflammation, and/or asthma. It would be worth trying to work not only on the most significant cells, that is, basophils, but also on eosinophils and neutrophils, without forgetting natural killer cells. Working with flow cytometry on inner and outer pollen subparticles seems to be of particular high interest.

*(2) Experimental Thunderstorm Studies*. If the electrostatic charge, less than 1 femtocoulomb, of pollen from different anemophilous plant species has already been measured [283], nevertheless, in the* polluen* field, very few studies are available on atmospheric electricity parameters and their role in this specific aerosol fraction. However, it seems quite relevant to better understand what could be, for instance, the impact of changes in the electric field or modifications of conductivity levels or vertical potential gradient changes. But, taking the things from a reverse side, it could be also interesting to understand the role of these very important particles' mass on the atmospheric electricity, when it is known that pollen can, for instance, play a significant role as giant cloud condensation nuclei in clouds formation [284]. Several initial trials were performed on this subject in the 1990s [285–287]. A more recent study gave insights on accumulation of charge by atmospheric particles (namely, starch granules from inner pollen subparticles) and deposition efficiency in the lower airway regions of the human respiratory tract [288]. The thunderstorm atmospheric conditions are of course quite special, with high O_3_ levels, specific moisture, high wind speeds, and electricity. It might be difficult to precisely mimic experimentally such complex conditions; nevertheless, it should be possible to get at least some close chamber conditions for studying* polluen* material and, later on,* polluen* effects.

*(3) About Rain*,* Fog*,* and Mist*. If sudden temperature changes as well as barometric ones seem of importance for pollen release and* polluen* interactions, water is also a key point. It plays, for instance, a noticeable role in inner pollen subparticles dispersion. Schäppi et al. evaluated that the proportion of dissemination of this specific fraction was of 37% in dry conditions and of 57% in case of light rain [113]. At the same time, in experimental conditions, Suphioglu et al. showed that the nebulization process could be of importance in asthma induction [118]. If works of Wang's team insisted also on the very important role of water and hydration in the* polluen* field, it should be quite relevant to better know the level of pollutants in rain water of different regions, in connection with bioaerosols load. Various PAHs present in rain, snow, and fog at specific levels had already been measured from place to place [289–291]. But what about the interactions of, for instance, “rainy” nanoparticles of ROFA, volatile organic compounds (VOCs), or aldehydes with wettable surface of pollen and/or pollen issued material fragments? At the same time, production of nanosized fine particles such as fullerene, titanium dioxide, and carbon nanotubes has increased rapidly in recent years. Large quantities of these fine particles are discharged to the environment intentionally or unintentionally in the course of their production, use, and disposal. What about the interactions of these other nanoparticles with pollen material in rainy situations [148]? Because an increase in humidity triggers a cascade of reactions resulting in activation of highly dynamic metabolic processes as well as a rapid increase of new compounds and conformational modifications of the pollen proteins content, studying rain, mist, and fog interactions sounds justified.

*(4) Molecular and Proteomic Approaches*. Through Bryce et al.'s work [96] and some others from the ZAUM group, it has been shown that allergenicity is determined by more than the sole allergen content. Nevertheless, molecular and proteomic approaches could still give rich elements in* polluen* studies. Indeed, allergens, as airborne proteins, may be structurally altered by air pollution. Some posttranslational modifications (PTMs) can affect their activity, conformation, folding, distribution, stability, and therefore function. They include glycosylation, phosphorylation, formylation, hydroxylation, methylation, S-nitrosylation, oxidation, and ubiquitination [154]. Providing experiments on these PTMs could be useful for the prediction of the increase of the allergic risk for an allergic patient to be in a polluted area. The expected effect of artificial pollution might be either qualitative, that is, associated with a decreased or higher number of allergens recognized in the polluted pollen extract, or quantitative, that is, a lower or a stronger binding to the polluted allergens than to the nonpolluted ones. The results could help clinicians anticipate and prevent the issue, especially in case of a suspected exacerbation, of clinical symptoms by recommending an adapted treatment.

The specific study of PTMs can be realized either directly from 2D gel electrophoresis analysis or after purification of the protein by immunoaffinity. It has been shown that the two ionization modes ESI and MALDI give complementary information [292]. Label-free approaches such as Multiple Reaction Monitoring should also be considered for the detection of specific modifications in complex peptidic mixtures [293]. Apart from physicochemical changes, air pollutants could have direct effects on IgE reactivity of allergens. It can be studied by immunoprint techniques after 2D gel separation of pollen protein extracts. Interactions between IgE and allergens (exhibiting or not pollutants induced PTMs) are thus to be considered at molecular level. A functional comparison between polluted and nonpolluted extracts regarding their ability to induce basophils degranulation is of obvious relevance.

Among the plant allergens currently described and listed by the International Union of Immunological Societies, around 25% are issued from pathogenesis-related proteins (PR) [294]. These PR proteins display multiple effects within the plant and can be regarded as a part of the plant's defense system [295]. It seems of evidence that when studying interactions of* polluen* with allergic people, they have to be envisaged among the most important ones.

The role of lipids in allergenicity is still understudied [296, 297]. The interactions of pollutants-lipids-allergens could be envisaged associating lipidomic and proteomic approaches. Finally, the last example that we would give of potential molecular works on* polluen* concerns epigenetic changes that occur on pollen material through diverse factors, for example, anthropogenic factors, and therefore that could play a role in allergic sensitization. It can have also consequences on allergy diseases and asthma [298] but, as far as we know, such research branch is still in infancy.

*(5) Multiple Oxidations*. When looking at the very rich work by Shalaby et al. [190], one can be eager to do the same but with polluted pollen material. Indeed, at the cross of oxidative stress and TLR4 activation and its associated TIR domain initiating the signaling cascade (in this case, TRIF), this team, from McGill University (Montreal), had in mind seeing if the protease activity of pollen (from* Betula populifolia* in this specific study) is capable of triggering TLR4-TRIF pathways and the development of allergic airway disease. The hypothesis was that oxidative stress, potentially activating the TLR4-TRIF pathways, is important in mediating allergic sensitization* via* the airway mucosa. TLR4 (Toll-like receptor 4 also known as CD284) not only is implicated in pathogen recognition and activation of innate immunity but also plays a very significant role in compromising mucosal tolerance to elicit allergic sensitization and/or to drive inflammatory responses to antigens* via* the airways.

After experimenting on BALB/c mice (TLR4- and TRIF-deficient ones as well as wild-type as controls), their conclusions were as follows. (i) Oxidative stress is important in amplifying airway disease independently of sensitization. (ii) Oxidative stress is critical to the development of airway inflammation consequently to inhaled pollen extract, while TLR4 and TRIF signaling are not necessary for mucosal sensitization to pollen leading to airway disease but modulate the inflammatory response. (iii) Pollen-induced oxidative stress is not critical for allergic sensitization. (iv) Intrinsic NAD(P)H oxidase activity, or other enzymatic activity, is not in the general pattern of pollen allergenicity. Thus, actively working on oxidative stress sounds crucial in the field of* polluen* studies.

## 6. Conclusions

Atmospheric pollution has direct effects on physical, chemical, and biological properties of the pollen grains. A large number of laboratory researches on artificial pollution of pollen as well as studies on pollen sampled in more or less polluted zones have clearly shown several noxious effects on pollen grains. As a first undoubted clue, for a large number of plant species, the viability and germination of pollen grains are modified by air pollutants, even at very low doses of exposure. The chemical composition of pollen is also modified by air pollution. Changes in inorganic ions composition are the most abundantly studied and documented chemical effect. Because of high NO_2_ content in polluted areas, nitration was documented and results showed that pollen proteins could be nitrated by urban or industrial pollution. But, at the same time, the ratio of nitrated pollen proteins in atmospheric conditions, whatever polluted or unpolluted ones, stays unknown. Moreover, interactions between pollen and air pollutants could lead to the formation of degraded components playing the role of adjuvant on the allergic response through essentially proinflammatory properties. Pollen is not equal in front of the pollution and there is a growing body of evidence showing that susceptibility of pollen to pollutants varies according to the plant species. It is also reported that pollen is more resistant when exposed* in vivo* rather than* in vitro*. Polluted pollen which may be called “*polluen*," because of the interface between pollutants and pollen, exhibits physical degradations of the exine, but the statistical significance of these observations is not known: what is the proportion of pollen grains showing these degradations in the atmosphere? These degradations lead to the release of pollen subparticles that were shown to contain allergens. The bioavailability of allergens increases as smaller particles penetrate more deeply in the respiratory tract. However, the effect of atmospheric pollution on the dispersion and allergenicity of these subparticles is for the moment still not clearly and not fully elucidated.

More studies are required in order to better understand the mechanisms of* polluen* rupture, its real occurrence in atmospheric conditions, and consequences on allergenic sensitization and immune and inflammatory boosting effect. Allergenic potential of pollen, quantified most frequently by the total major allergen content or skin prick test, is modulated by atmospheric pollution. In a majority of plant species,* polluen* is more allergenic than healthy pollen even though some controversial results have been reported.

On animal models, whatever aspect is considered, gases, PM, DEP, or combined pollutants effects, no clear and unambiguous lines of evidence can be put forward. With some pollen sources, some specific animals, and some pollutant levels, IgE concentration can change (polluted versus natural control). The change in IL5 level, one of the “allergic” Th2 cytokines, has been demonstrated in some cases. Similarly, nonunivocal results were obtained in* in vivo* human experiments and with human cells (*in vitro* and* ex vivo*). Based upon several experimental reports, some consensus nevertheless emerged. For instance, increasing DEP with constant levels of allergen resulted in increasing clinical prevalence of allergy at respiratory level. Also, whatever the type of cells (neutrophils, basophils, or epithelial and dendritic cells) polluted material has more effects than unpolluted control one. Obviously allergenicity is a plastic notion depending not only on intrinsic characteristics of the allergenic source such as its physical nature (molecule, particle, organism, etc.), its quantitative environmental level, and its route of penetration into humans but also on the biogenic associated molecules (bacteria, virus, lipids, and organic small molecules) and the xenogeneic and anthropogenic associated pollutants (gases, PM, etc.). All these various partners interact together at different levels of the sensitization process, from the contact with the first barrier defense (skin and nasal or bronchial mucosa) to the cellular activation in tissue, blood stream, and lymph nodes. Interacting partners are multiple as well as tissue and cellular targets. Some are still unknown as, for instance, cellular targets for gases such as NO which is at the crossroad of various activation pathways. Deciphering all triggering signals is a challenge for the next future and will require relevant and efficient analytic and synthetic tools.

At an environmental level, there is nowadays no quantitative indicator to evaluate the extent of pollution suffered, intrinsically and/or atmospherically, by a pollen grain in “real life.” To better understand the complex species-dependent interactions between plant, soil, atmosphere, and meteorological conditions as well as the effect of these interactions on health, more systematic interdisciplinary standardized studies are needed, which should use, before all, well-described pollen grains (in terms of species, sampling, storage conditions, macromolecules, and water content) and well-characterized pollutant doses.

## Figures and Tables

**Table 1:** Studies of the effect of different air pollutants on birch pollen grains.

Birch species	Pollutant(s) studied	Concentration range	Duration of exposure	Germination or viability	Total proteins	Protein profiles	IgE recognition	Reference
*B. pendula *	NO_2_	34/67 ppb	6/48 h	↘	↘	—	↗	[153]
*Birch allergen*	NO_2_	—	—	—	—	—	↗	[154]
*B. verrucosa*	NO	1–5%	48 h	—	↘	Numerous modifications	—	[155]
*B. alba*	NO_2_	1–400 ppm	Hours to days	—	—	—	—	[134]
*B. pendula*	O_3_	61–192 ppb	6/12 h	↘	↘	—	—	[156]
*B. papyrifera*	O_3_	Ambient/ambient × 1.5	9 years	↘	—	—	—	[87]
*B. pendula*	CO	10–31 ppm	6/12 h	↘	↘	—	—	[156]
*B. papyrifera*	CO_2 _	360–560 ppm	9 years	↗	—	—	—	[87]
*B. verrucosa*	CO	0.01–5%	48 h	—	↘	Numerous modifications	—	[143, 155]
*B. verrucosa*	CO	100%	2 h	—	—	—	—	[69]
*B. pendula*	Comparison rural/urban	—	—	—	—	↗ Bet v 1	—	[172]
*B. pendula*	SO_2_	130–540 ppb	6/12 h	↘	↘	—	—	[156]
*Betula *sp.	NO_2_/O_3 _/urbanization index	—	—	—	—	↗ (Bet v 1 for O_3_)	↗ (for O_3_)	[98]
*Betula *sp.	Comparison rural/urban	—	—	—	—	Differences in spot intensities	—	[96]
*B. papyrifera*	CO_2_ + O_3_	CO_2_: 360–560 ppm O_3_: Ambient/ambient × 1.5	9 years	=	—	—	—	[87]
*Birch (proteins)*	Urban air or NO_2_ + O_3_	100 ppb	5/50 h	—	—	Nitration of Bet v 1	—	[173, 174]
*B. pendula, B. pubescens*	Sulfur and heavy metals	—	—	—	=	—	**=**	[55]
*B. verrucosa*	Comparison rural/urban	—	—	↘	—	—	—	[80]
*B. verrucosa*	SO_2_	1%	2 h	—	—	—	—	[69]
*Betula *sp.	Urban with traffic	Exposed to urban pollution	24/72 h	—	—	—	—	[66]

**Table 2:** Studies of the effect of different air pollutants on grass pollen grains.

Grass species	Pollutant(s) studied	Concentration range	Duration of exposure	Germination or viability	Total proteins	Protein profiles	IgE recognition	Reference
*Dactylis glomerata, Phleum pratense *	NO_2_	2000 ppb	4 h	—	=	—	↘	[175]

*Festuca elatior*	NO_2_	10,000 ppm	3 min	—	=	Changes observed	—	[141]

*Phleum pratense *	O_3_	30–80 ppb	Plant life	—	—	↘ Phl p 5	—	[176]

*Dactylis glomerata, Phleum pratense *	O_3_	100 ppb	4 h	—	=	—	↘	[175]

*Lolium perenne*	O_3_	60 ppb	2 weeks	—	↗	↗ Lol p 5	—	[99]

*Lolium perenne*	O_3_	2 outdoor sites		—	—	↗ Lol p 5	—	[99]

*Phleum pratense *	CO_2_	400–800 ppm	Plant life	—	—	=	—	[176]

*Dactylis glomerata *	NO or CO	1–5%	48 h	—	↘	Numerous modifications	—	[155]

*Dactylis glomerata *	CO	0.01–5%	48 h	—	↘	Changes observed	—	[143]

*Festuca elatior*	CO	10,000 ppm	3 min	—	=	Changes observed	—	[141]

*Phleum pratense*	SO_2_	13 mg/m^3^	18 h	—	—	—	=	[177]

*Dactylis glomerata, Phleum pratense *	SO_2_	2000 ppb	4 h	—	=	—	↘	[175]

*Festuca elatior*	SO_2_	10,000 ppm	3 min	—	=	Changes observed		[141]

*Phleum pratense *	Toluene, m-xylene	125 mg/m^3^	18 h	—	—	↗ Phl p 5	↗	[177]

*Dactylis glomerata, Phleum pratense*	O_3_/NO_2_	100–2000 ppb	4 h	—	=	—	↘	[175]

*Dactylis glomerata, Phleum pratense *	NO_2_/SO_2_	2,000 ppb each	4 h	—	=	—	↘	[175]

*Lolium perenne*	Rural/urban	—	—	—	↗	Different patterns	↗	[56]

*Phleum pratense *	Atmospheric particulate matter	Not mentioned	4 h	—	↗	=	↗	[136]

*Dactylis glomerata*	Road traffic			↘	—	—	—	[88]

*Dactylis glomerata *	Car exhaust	10–60 min	10–300 min	—	↘	=	↘	[149]

*Dactylis glomerata *	Cigarette smoke	100 HP	1–28 days	—	↘	=	↘	[149]
